# A Novel Hepatocellular Carcinoma Image Classification Method Based on Voting Ranking Random Forests

**DOI:** 10.1155/2016/2628463

**Published:** 2016-05-17

**Authors:** Bingbing Xia, Huiyan Jiang, Huiling Liu, Dehui Yi

**Affiliations:** ^1^Software College, Northeastern University, Shenyang 110819, China; ^2^The Department of Hepatobiliary Surgery, The First Affiliated Hospital of China Medical University, Shenyang 110001, China

## Abstract

This paper proposed a novel voting ranking random forests (VRRF) method for solving hepatocellular carcinoma (HCC) image classification problem. Firstly, in preprocessing stage, this paper used bilateral filtering for hematoxylin-eosin (HE) pathological images. Next, this paper segmented the bilateral filtering processed image and got three different kinds of images, which include single binary cell image, single minimum exterior rectangle cell image, and single cell image with a size of *n*⁎*n*. After that, this paper defined atypia features which include auxiliary circularity, amendment circularity, and cell symmetry. Besides, this paper extracted some shape features, fractal dimension features, and several gray features like Local Binary Patterns (LBP) feature, Gray Level Cooccurrence Matrix (GLCM) feature, and Tamura features. Finally, this paper proposed a HCC image classification model based on random forests and further optimized the model by voting ranking method. The experiment results showed that the proposed features combined with VRRF method have a good performance in HCC image classification problem.

## 1. Introduction

As we all know, liver cancer is a common disease and, just like some other diseases, the global morbidity and mortality rates have significant increases recently. This problem is a huge challenge that mankind will face for a long time [1, 2]. The main clinical characteristics of liver cancer are low predictability, quick deterioration, and being more susceptible to death after getting the cancer. Therefore, the early diagnosis and treatment of liver cancer are significantly important for patient, which also increase the possibility of cure.

Pathological image plays a decisive and irreplaceable role in the liver cancer diagnosis; at the same time, the rise of Computer Aided Diagnosis (CAD) also brings a new breakthrough for the diagnosis and treatment of liver cancer [3]. The use of image analysis for pathological images treatment has huge advantages; for example, the computer can find the subtle variations in pathological image. Besides, it can take the place of a doctor to do repetitive work. In a word, CAD has an extremely important research meaning and practical value for clinical hepatocellular carcinoma (HCC) image classification and diagnosis.

With the rapid development of computer technology and the rise of Computer Aided Diagnosis, a large number of scholars and specialists have participated in HCC image classification and yielded many fruits. However, most of the researchers mainly focus on liver CT image classification and recognition. Downs et al. [4] proposed a decision support tool based on fuzzy ARTMAP for the diagnosis of breast cancer. This method solved the subjective inaccuracy cognizance problem with objective fuzzy category membership by using fuzzy recognition theory and finally realized HCC image diagnosis. Schnorrenberg et al. [5] proposed a novel breast cancer cell artificial neural network (ANN) classifier based on feedback neural networks, and the ANN method has self-learning, self-adaption, self-organization, and massively parallel processing ability. Blekas et al. [6] also used fuzzy pattern recognition and combined morphometry with fuzzy maximum neural network classifier and designed a diagnosis system for recognizing the benign and malignant neoplasias. Mat-Isa et al. [7] proposed a feature extracted method based upon region growing and a novel ANN method, which realized the recognition and grading for cervical carcinoma cell image.

This paper proposed a novel random forests classification model based on voting ranking method; besides, this paper defined three innovative atypia features including auxiliary circularity, amendment circularity, and cell symmetry. At the same time, this paper creatively proposed center-proliferation segmentation (CPS) method and got three kinds of single cell image including single binary cell image (SBCI), single minimum exterior rectangle cell image (SMESRCI), and single cell image with a size of *n∗n* (SNSCI). In the experiment, this paper adopted 10-fold cross-validation method for testing the proposed method VRRF, and this paper compared the VRRF with support vector machines (SVM), *k*-nearest neighbor (kNN), and conventional random forests (RF). The experiment results showed that the proposed atypia features combined with some other features are useful for the pathological character of single liver cell and HCC expression. Meanwhile, the proposed VRRF method showed good performance in hepatocellular carcinoma image classification and strong robustness.

## 2. Hepatocellular Carcinoma Image Classification Method

**Figure 1:**
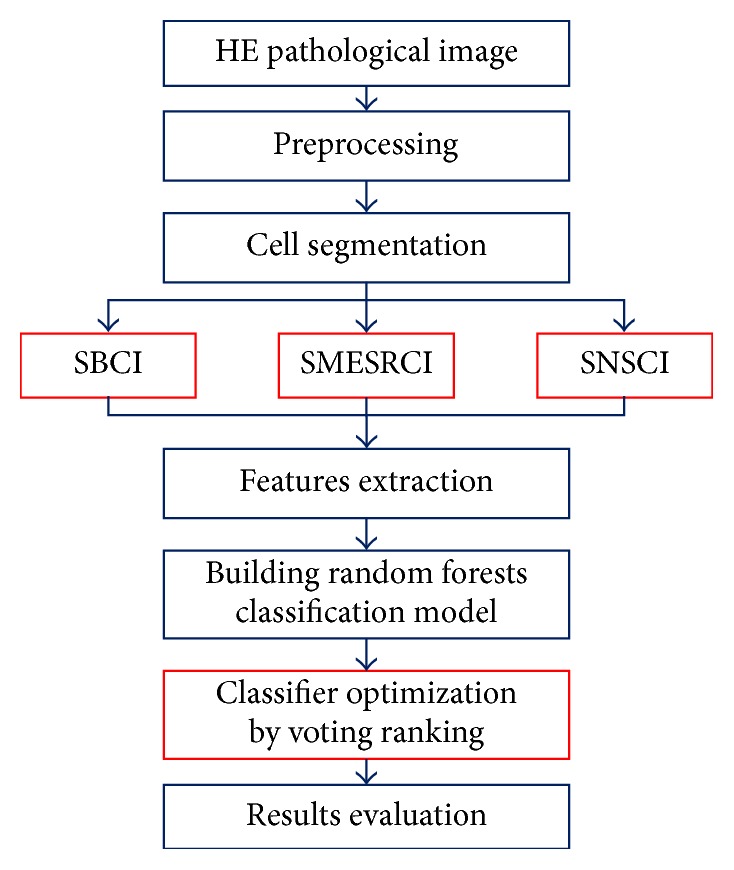
The flowchart of this paper proposed HE pathological image classification method.

The whole work of hepatocellular carcinoma image classification method includes using bilateral filtering for image preprocessing firstly. Then, after segmentation, this paper got three kinds of single cell images like single binary cell image, single minimum exterior rectangle cell image, and single cell image with a size of *n∗n*. After that, this paper creatively proposed atypia features which include auxiliary circularity, amendment circularity, and cell symmetry. Besides, this paper extracted some shape features, fractal dimension features, and several gray features based on gray-scale image, for instance, Local Binary Pattern (LBP) feature, Gray Level Cooccurrence Matrix (GLCM) feature, and Tamura features. Next, this paper divided the image data into training set and testing set; at the same time, this paper also gave every single image a label. Finally, this paper proposed a novel voting strategy in random forests classification's voting stage which has been named voting ranking. The method's flowchart proposed by this paper is shown in Figure 1.

In Figure 1, the red rectangular frame means the innovation work of this paper. Besides, in the figure, SBCI, SMESRCI, and SNSCI are three kinds of single cell images; SBCI represents single binary cell image; SMESRCI means single minimum exterior rectangle cell image; and SNSCI is single cell image with a size of *n∗n*, respectively.

### 2.1. Preprocessing

This paper used HE dying HCC image for classification work. Because of the dye difference of pathological image caused by the difference of dye and the dyeing time, this paper gave up using the color features.

**Figure 2:**
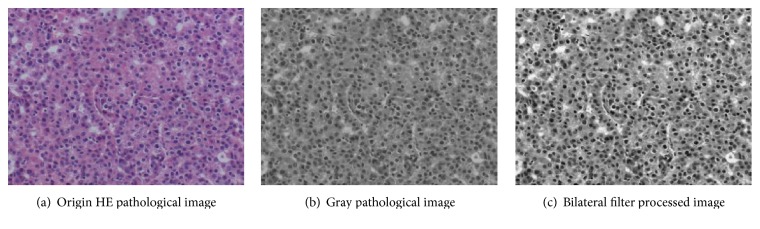
Bilateral filter processed results image.

Before segmentation, the origin image may have lots of noise information. Bilateral filter proposed by Tomasi and Manduchi [8] can process the whole image at a time. For image denoise processing, bilateral filter not only can realize image denoising but also can save the high frequency information of origin image, which is particularly important. This paper used bilateral filter realized image denoise and enhancement; the results are shown in Figure 2(c).

### 2.2. Cell Segmentation

In this paper, cell segmentation step includes coarse segmentation and single cell image segmentation. The single cell image segmentation was totally proposed in this paper and named center-proliferation segmentation (CPS) method. In coarse segmentation step, segment the bilateral filter processed image and get binary coarse segmentation result. After that, in CPS step, get three kinds of single image which include single binary cell image, single minimum exterior rectangle cell image, and single cell image with a size of *n∗n*. The details of these two segmentation methods will be introduced in the following section.

#### 2.2.1. Coarse Segmentation

**Figure 3:**
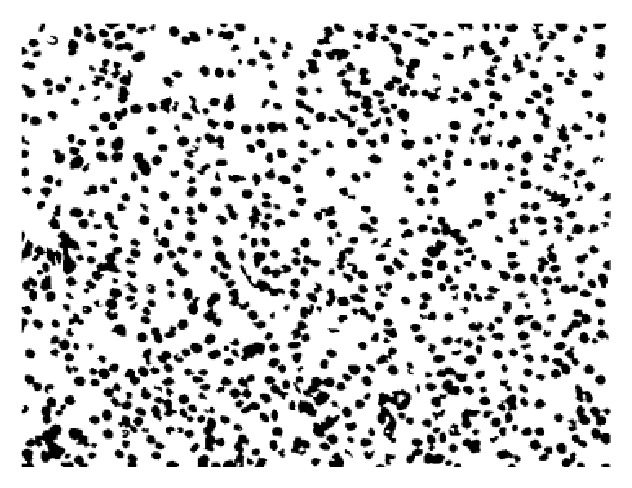
The binary cell coarse segmentation result.

Firstly, for the bilateral filter processed image in Figure 2(c), this paper used normal threshold realized coarse segmentation for binary image and did some morphological correction works; the final coarse results are shown in Figure 3.

#### 2.2.2. Center-Proliferation Segmentation (CPS) Method

**Figure 4:**
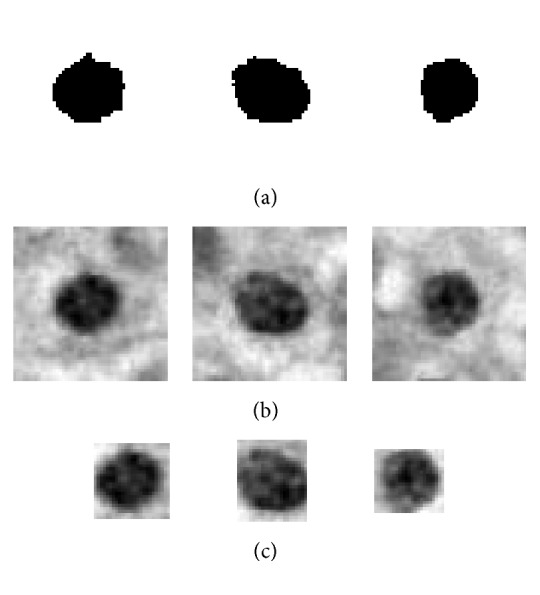
The three different kinds of segmentation results. (a) is single binary cell image, (b) is single cell image with a size of *n∗n*, and (c) is single minimum exterior rectangle cell image.

For the binary coarse segmentation result as shown in Figure 3, this paper adopted center-proliferation segmentation (CPS) method and detailed steps are shown in Algorithm 1 and the segmentation results of Algorithm 1 are shown in Figure 4.

**Algorithm 1 (center-proliferation segmentation (CPS) method).**

  

*Input*. The input is the HE pathological image coarse segmentation results.

*Output*. The output is the single binary cell image (SBCI), single minimum exterior rectangle cell image (SMERCI), and single cell image with a size of *n∗n* (SNSCI).

*Steps*

(1) Get the connected region in Figure 3.
(2) Compute the connected region's circularity and select the connected region by circularity threshold value which is set to a value greater than 0.85 in this paper.
(3) For the saved connected region in Step (2), get *n∗n* pixel size binary square block from the center to the four directions which can eliminate the effect of image size difference. Several experiments showed that *n* = 51 is the best. Finally, save the 51*∗*51 pixel size image.
(4) There may be two or more connected regions in single *n∗n* pixel size binary square block; for this situation, only reserve the specific region which is located in the center of image; at the same time, get the center point coordinate of it.
(5) Map the center point coordinate which is gotten in Step (4) into the gray image as shown in Figure 2(b). Then, get *n∗n* pixel size gray square block from the center to the four directions like Step (3). There may be two or more cells in one gray image block, and this is one problem which should be solved in future work.
(6) For the single binary cell image in Step (4), compute the minor axis and major axis; then, this paper got the bounding rectangle gray image using the same way in Step (5).

### 2.3. HCC Feature Extraction

For every kind of single cell image, this paper extracted different features, respectively. First of all, this paper extracted shape features and fractal dimension features for single binary cell image (SBCI) as shown in Figure 4(a). Then, for the single cell image with a size of *n∗n* (SNSCI) as shown in Figure 4(b), this paper extracted gray entropy features, the mean and standard deviation of image gray value, LBP feature, GLCM feature, and Tamura features. Finally, this paper creatively proposed three atypia features including auxiliary circularity, amendment circularity, and cell symmetry. All of these features are extracted or proposed in this paper as shown in Table 1, and the italic font means the features proposed by this paper.

#### 2.3.1. Atypia Features

Because of the complexity of HCC cell image, the common features cannot show its characters very well. This paper creatively proposed the atypia features of HCC cell image which aimed to describe HCC cell images' morphological characteristics characters as clearly as possible. The proposed atypia features include auxiliary circularity, amendment circularity, and cell symmetry.

Firstly, the computational formula of auxiliary circularity is shown in (1) and the amendment circularity is shown in (2):

(1) Cf=4πA6.8/A+0.625L2,

(2) Cw=4Aπw2+h2,

where *C*_*f*_ is the auxiliary circularity and (6.8/*A* + 0.625) is the correction factor based on original circularity. *C*_*w*_ is the amendment circularity which means the radio of nuclear area and outer circle area. *A* means the area of cell in the image, *L* means the perimeter, and *w* and *h* are the width and length of the external rectangle, respectively.

**Figure 5:**
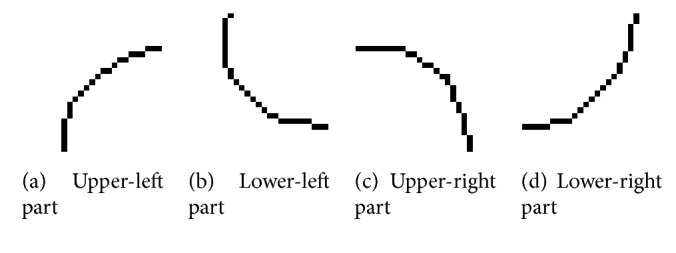
The four-part segmented results image of SBCI after edge detection by Canny operator.

Secondly, the cell symmetry feature which used fractal dimension calculation will be introduced in the following section. Here, achieve boundary detection for the single binary cell image (SBCI) by Canny operator. After that, segment the boundary detection results in horizontal and vertical direction and finally get four-part images; the results are shown in Figure 5.

For the four parts given by Figure 5, firstly, calculating fractal dimension, respectively, and getting *F*_*a*_, *F*_*b*_, *F*_*c*_, and *F*_*d*_, the specific calculation method of fractal dimension will be introduced in the following section. This paper defined the cell symmetry of SBCI by calculating the sum of the difference between two adjacent parts of fractal dimensions' absolute value as shown in

(3) $$\begin{array}{c} S_{ym}=\left|\;F_{a}-F_{b}\;\right|+\left|\;F_{a}-F_{c}\;\right|+\left|\;F_{b}-F_{d}\;\right|+\left|\;F_{c}-F_{d}\;\right|, \end{array}$$

where S_ym_ means the symmetry of single binary cell image (SBCI) and *F*_*a*_, *F*_*b*_, *F*_*c*_, and *F*_*d*_ mean the fractal dimension of four parts in Figure 5. As we all know, when the cell is nearly circular, the difference of adjacent parts' fractal dimensions is close to 0. So if the symmetry of cell is more symmetrical, S_ym_ will be smaller.

#### 2.3.2. Other Features

First of all, for single binary cell image (SBCI), this paper extracted shape features which include area, perimeter, roundness, elongation, and rectangularity. Because the method of the shape features' extraction is simple, this paper does not explain it too much.

Then, this paper extracted fractal dimensions features. For fractal dimension, as we all know, the simple object can be described by the traditional geometric method, but most objects' real shape is quite complex and can not be described by traditional geometric method very well. So Stach and Cybo [10] established the fractal geometry theory which can measure the natural objects' irregularity. The fractal dimension features used in this paper combined single fractal dimension with multifractal dimensions.

This paper's multifractal dimension calculation used the method proposed by Posadas et al. [11] and the single fractal dimension calculation used the method proposed by Chaudhuri and Sarkar [12]. The single fractal dimension adopted boxing counting algorithm and the initial boxing size is the 1/10 length of the image. For multifractal dimension theory, it established the relationship between the local scale characteristic and the general character of the fractal object. For single binary cell image (SBCI), first of all, achieve boundary detection by Canny operator and then calculate single fractal dimension and multifractal dimensions. Some of the fractal dimension calculation results are shown in Table 2.

Besides, this paper extracted several kinds of gray features which include gray entropy features, the mean and standard deviation of image gray value, LBP feature, GLCM feature, and Tamura features for single cell image with a size of *n∗n* (SNSCI) and single minimum exterior rectangle cell image (SMERCI) as shown in Figures 4(b) and 4(c). The specific introduction of gray features is shown in the following.

*(1) The Mean and Standard Deviation of Image Gray Value.* For the computation of the mean and standard deviation of image gray value, we computed every single pixel's gray value instead of the whole images. For the mean of image gray value, it can represent the whole image's gray shade; the standard deviation of image gray value means the nonuniformity of gray distribution in one image. In this paper, for SNSCI, compute the mean and standard deviation of image gray value.

Comparing cancerous cell image with normal liver cell image, the former one has deeper dye and presents uneven dyeing. So the mean of cancerous cell image gray value is smaller than the normal one, but the standard deviation of cancerous cell image gray value is bigger than the normal one. These two characters can be used for HCC image classification very well. 

*(2) Gray Entropy*. The image gray entropy is useful feature, and the gray entropy can reflect the average information of an image. In this paper, adopt first-order gray entropy which can reflect the amount of information included in the accumulation characters of image gray distribution. The definition of gray entropy is shown in

(4) $$\begin{array}{c} H=-\overset{255}{\underset{i=0}{\sum }}p_{i}\log p_{i}, \end{array}$$

where *p*_*i*_ is the probability of one gray-scale to occur in one image. 

*(3) Local Binary Pattern (LBP) Feature*. LBP feature firstly is proposed by Ojala et al. [13] in 1996; it is a kind of operator which can be used to describe image pattern textural feature, and it has several advantages such as rotational invariance and gray-scale invariance. For SNSCI and SMERCI, the LBP feature extraction method is shown in Algorithm 2 [13].

**Algorithm 2 (Local Binary Pattern feature extraction algorithm).**

  

*Input*. The input is SNSCI and SMERCI.

*Output*. The output is LBP feature.

*Steps*

(1) Divide the input image into *n∗n* size cell, and this paper sets value *n* to 3.
(2) For the every pixel in the cell, compare its gray value with neighboring 8 pixels' gray value. If the neighboring pixel's gray value is bigger than the center pixel's gray value, then mask the neighboring pixel as 1 or mask it as 0. So in this 3*∗*3 cell, generating 8-bit binary codes, it can be similar to LBP value of cell's center pixel.
(3) Compute the histogram of each cell which also can be seen as computing the probability of each number (assume it as decimal number), and then normalize the histogram.
(4) Collect each cell's histogram into a feature vector, and it is the whole image's LBP textural feature.

*(4) Gray Level Cooccurrence Matrix (GLCM) Feature.* GLCM feature firstly was proposed by Haralick and Shanmugam [14] in 1973 which used to describe textural feature. In GLCM, angular second moment (ASM) can be used to reflect image gray distribution uniformity and textural detail; entropy (ENT) reflects the image gray distribution heterogeneity or complexity; contrast (CON) reflects the image clarity and texture depth; correlation (COR) can be used to reflect local gray correlation in image. So this paper generated 8 matrixes in 4 directions *θ* = {0°, 45°, 90°, 135°} and 2 distances *d* = {1,2}, using these 8 Gray Level Cooccurrence Matrixes to extract features. Besides, compute the mean and variance of ASM, ENT, CON, and COR on 2 distances and finally get 16 features. The computational formula of these four textural features is shown in

(5) ASM=∑i∑jIi,j2,ENT=−∑i∑jIi,jln⁡Ii,j,CON=∑i∑ji−j2Ii,j,COR=∑i∑ji,jIi,j−uxuyσxσy,

where *I*(*i*, *j*) is the *i*th row and *j*th column element and the definition of *u*_*x*_, *u*_*y*_, *σ*_*x*_, and *σ*_*y*_ is shown as follows: *u*_*x*_ = ∑_*i*_∑_*j*_ *I*(*i*, *j*), *u*_*y*_ = ∑_*i*_ *i*∑_*j*_ *I*(*i*, *j*), *σ*_*x*_ = ∑_*i*_(*i* − *u*_*x*_)∑_*j*_ *I*(*i*, *j*), and *σ*_*j*_ = ∑_*j*_(*j* − *u*_*y*_)∑_*i*_ *I*(*i*, *j*).

*(5) Tamura Feature*. In 1978, Tamura et al. [15] proposed the Tamura textural features by studying the texture of human visual perception's psychological research. Tamura features include coarseness, contrast, directionality, linelikeness, roughness, and regularity, and the former three features are specially important [16]. The computation method of Tamura features is given by [15]; this paper used former five features without regularity.

### 2.4. The Random Forests Based on Voting Ranking Random Forests (VRRF)

**Figure 6:**
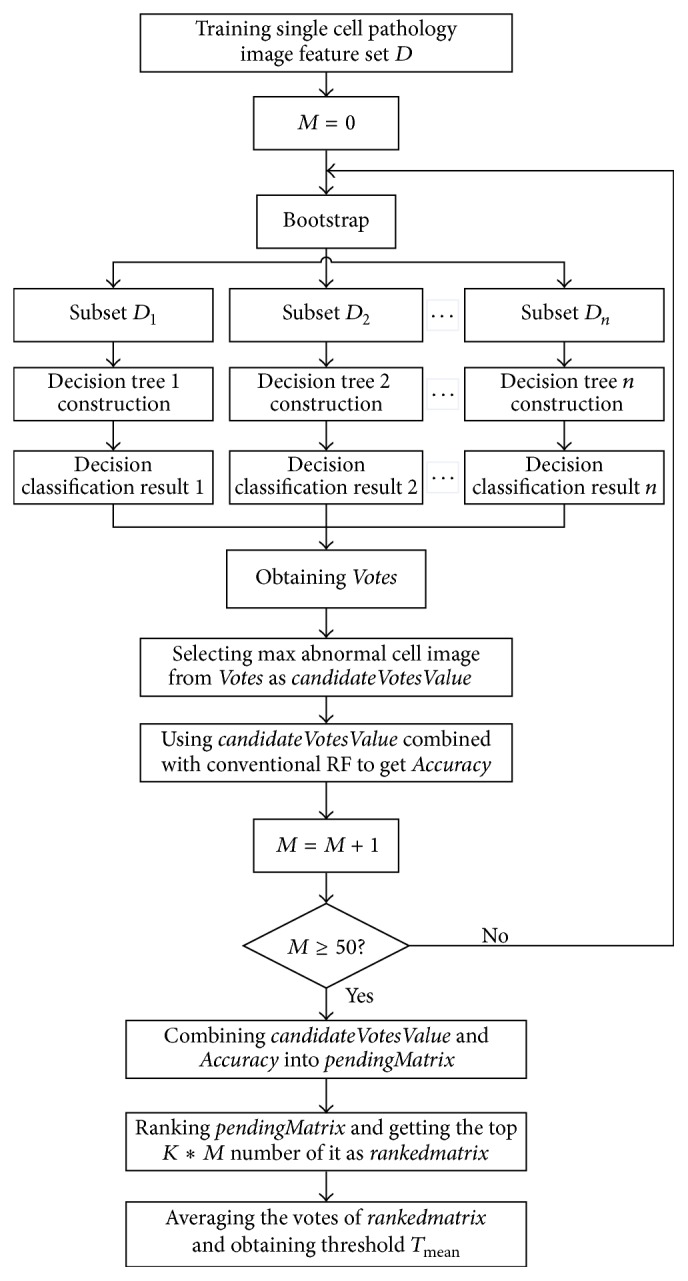
The flowchart of HCC image classification model based on voting ranking random forests (VRRF).

Based on the conventional random forests method, after getting the vote numbers of each training image by decision trees, this paper optimized the vote matrix and proposed the random forests classification method based on voting ranking random forests (VRRF). The VRRF model's implementation steps are shown in Algorithm 3 and the flowchart of HCC image classification model based on VRRF is shown in Figure 6.

**Algorithm 3 (random forests classification method based on voting ranking algorithm).**

  

*Input*. The input is the feature matrix extracted from SBCI SNSCI and SMERC.

*Output*. The output is the classification results.

*Steps*

(1) Input the extracted feature matrix and the corresponding label matrix labeled by the authorities.
(2) Get 500 data subsets from the training set and training label by Bootstrap process.
(3) Construct a decision tree by each data subset and obtain 500 decision trees finally.
(4) Collect the results of each decision tree and obtain a *N∗*2 size vote matrix* Votes*, where *N* is the number of input images. The first row of* Votes* is the votes of normal cell image and the second row is the votes of HCC image.
(5) Select the maximum vote value from the second row of* Votes* and consider it as a candidate vote which is named* candidateVotesValue*.
(6) Use the* candidateVotesValue* given in Step (5) instead of conventional random forests' default threshold value 250 and obtain the classification accuracy which is named* Accuracy*.
(7) Loop from Step (2) to Step (6) for *M* times (*M* = 50 in this paper) and obtain a *M∗*2 pending votes matrix which is named* pendingMatrix*. The first row of* pendingMatrix* is* candidateVotesValue* gotten in Step (5) and the second is the* Accuracy* gotten in Step (6).
(8) Rank the* pendingMatrix* according to the accuracy from high to low and obtain top *K∗M* votes from it as* rankedmatrix; K* means the percentage and it is set to be 0.3 in this paper according to the experience. Average the votes of* rankedmatrix* and obtain final threshold *T*_mean_ which is used to replace the fixed threshold in conventional random forests method.

## 3. Results and Discussion

### 3.1. Experimental Data and Platform

The experimental data are provided by the pathology department of a large hospital in Shenyang, China. After segmentation, the number of obtained training images and the number of testing images are shown in Table 3.

The experimental platform is Intel® Core*™* i7-2600 CPU @3.4 GHz, 8 G RAM, 900 G hard disk, Windows 7 OS, and MATLAB R2014a simulation environment.

### 3.2. Experimental Evaluative Criteria

This paper used accuracy (ACC), sensitivity (SEN), and specificity (SPE) to evaluate classification performance of VRRF. All the labels of training data and testing data are given by experts in pathology department, so it has certain professional authority. The definitions of the evaluation criterion are shown in

(6) ACC=TP+TNTP+FN+TN+FP,SEN=TPTP+FN,SPE=TNTN+FP,

where TP and FN are the number of HCC cell images which were correctly classified and incorrectly classified, respectively. TN and FP are the number of the normal liver cell images which were correctly classified and incorrectly classified, respectively. Sensitivity indicates the proportion of HCC cell images that are correctly classified and specificity indicates the proportion of normal cell images that are correctly classified.

### 3.3. Experimental Results Analysis

**Figure 7:**
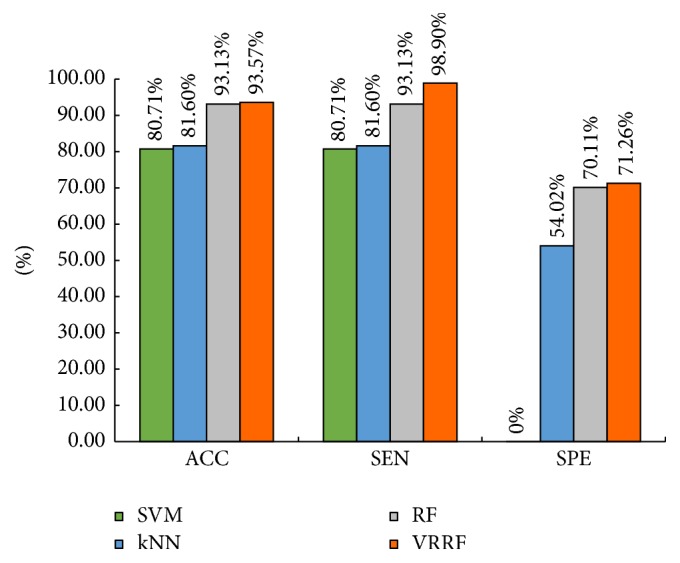
The performance comparison between SVM, kNN, RF, and VRRF.

To evaluate the effectiveness of the proposed VRRF algorithm, this paper compared the VRRF with SVM, kNN, and RF; the evaluation criteria include accuracy, sensitivity, and specificity. The experimental data of the comparison experiment are shown in Table 3. In VRRF method, according to several experiments, *K* = 0.3, in Algorithm 3, Step (8) of this paper.  Figure 7 gives the intuitive comparison of these four classifiers.

Comparing to SVM or kNN, the proposed VRRF method is better in accuracy, sensitivity, or specificity as shown in Figure 7. When it comes to conventional random forests, the proposed VRRF method also has a certain improvement. But the specificity of the proposed VRRF method still remains to improve and this is also a part of future work.

At the same time, this paper did 10 times 10-fold cross-validation and the results are shown in Table 4. The final accuracy of the proposed VRRF method is the mean value of 10 times cross-validation results.

**Figure 8:**
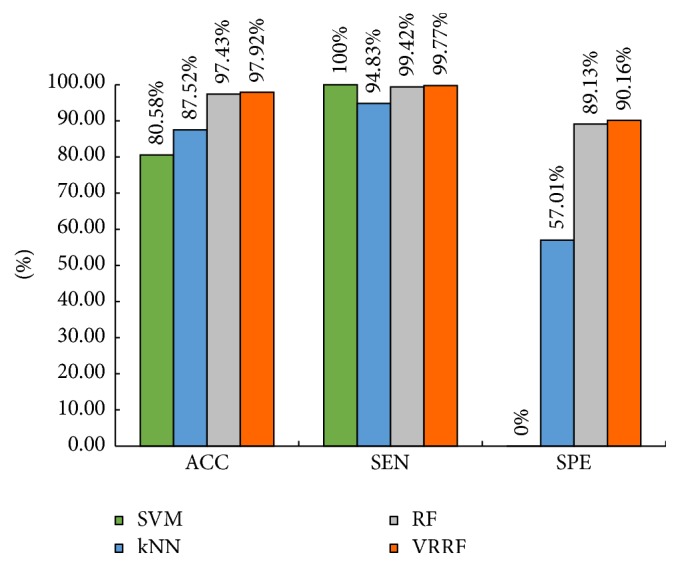
The performance comparison between four classifiers combined with 10-fold cross-validation.

According to the results of 10-fold cross-validation given by Table 4, this paper did a performance comparison of all four classifiers combined with 10-fold cross-validation, and the comparison results are shown in Figure 8.

The proposed VRRF method adopted voting ranking strategy; comparing to the fixed threshold of conventional RF, it can choose the votes which respond to the relatively high classification accuracy and finally get a more rational threshold. As we can see, in Figure 8, after 10-fold cross-validation, comparing to SVM, kNN, and conventional RF, the proposed VRRF method is the best among all four classifiers in accuracy, sensitivity, or specificity.

## 4. Conclusion

This paper proposed a classification method for three kinds of segmented single cell image, which include single binary cell image (SBCI), single minimum exterior rectangle cell image (SMERCI), and single cell image with a size of *n∗n* (SNSCI). For these kinds of image, this paper defined auxiliary circularity, amendment circularity, and cell symmetry. Furthermore, this paper extracted some shape features, fractal dimension features, and gray features. Besides, this paper proposed a novel random forests classifier based on voting ranking random forests (VRRF) for HCC cell image classification. The VRRF method is based on conventional random forests model and optimized the voting step later. This paper adopted 10-fold cross-validation and the experimental results showed that the proposed VRRF method has a better performance than SVM, kNN, or conventional RF.

Comparing to SVM and kNN, the conventional random classifier and the proposed VRRF method have higher time complexity; this is what this paper should improve in the future work. In addition, this paper would like to extract more useful features; at the same time, realizing HCC cell image multiclassification also is a necessary work in the future.

## Figures and Tables

**Table 1:** The features list.

Features type	Features name
*Atypia features*	*Auxiliary circularity, amendment circularity, cell symmetry*

Other features	
Shape features [9]	Area, perimeter, roundness, elongation, rectangularity
Fractal dimensions features	Single fractal dimension, multifractal dimensions
Gray features	The mean and standard deviation of image gray value, gray entropy, Local Binary Pattern (LBP), Gray Level Cooccurrence Matrix (GLCM), Tamura

**Table 2:** Some of the single binary cell images' fractal dimension calculation results.

SBCI	Single fractal dimension	Multifractal dimensions						
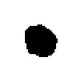	1.7255003105	−1.4090	1.2563	1.1403	0.1826	1.1659	1.1348	0.0490
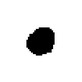	1.9008654554	−1.3471	1.1614	1.0743	0.1372	1.0904	1.0624	0.0442
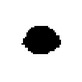	1.8295695208	−1.4327	1.1605	1.1226	0.0597	1.1378	1.1226	0.0236

**Table 3:** The number of images adopted in the experiment.

Image type	Training images number	Testing images number	Total images number
Normal single cell image	109	87	196
HCC single cell image	463	364	827

**Table 4:** The experiment results of proposed VRRF method combined with 10-fold cross-validation.

	Experiment times									
	First time	Second time	Third time	Fourth time	Fifth time	Sixth time	Seventh time	Eighth time	Ninth time	Tenth time
Onefold	1	0.8431	1	0.9608	0.9902	0.8824	1	1	1	1
Twofold	1	1	0.9903	1	1	0.8812	1	0.9505	1	1
Threefold	0.9903	0.9314	0.9902	1	0.9902	1	1	1	0.9406	0.9608
Fourfold	0.9804	1	0.9505	1	1	1	1	0.9903	0.8431	1
Fivefold	1	1	1	1	0.9903	0.9706	0.9208	0.9604	0.9902	0.9903
Sixfold	0.9307	0.9804	1	1	1	0.9902	1	0.9314	0.9902	1
Sevenfold	0.9903	1	1	0.8515	1	0.8911	0.9510	1	1	1
Eightfold	0.9703	1	1	0.9903	1	1	0.8713	0.9901	0.9903	1
Ninefold	1	0.9903	1	0.9902	0.9314	1	1	0.9902	1	1
Tenfold	1	1	1	0.9903	0.9903	1	0.8431	1	1	0.9902
